# Comparing Alternative Methods for Holding Virgin Honey Bee Queens for One Week in Mailing Cages before Mating

**DOI:** 10.1371/journal.pone.0050150

**Published:** 2012-11-16

**Authors:** Gianluigi Bigio, Christoph Grüter, Francis L. W. Ratnieks

**Affiliations:** Laboratory of Apiculture and Social Insects, School of Life Sciences, University of Sussex, Falmer, Brighton, United Kingdom; Arizona State University, United States of America

## Abstract

In beekeeping, queen honey bees are often temporarily kept alive in cages. We determined the survival of newly-emerged virgin honey bee queens every day for seven days in an experiment that simultaneously investigated three factors: queen cage type (wooden three-hole or plastic), attendant workers (present or absent) and food type (sugar candy, honey, or both). Ten queens were tested in each of the 12 combinations. Queens were reared using standard beekeeping methods (Doolittle/grafting) and emerged from their cells into vials held in an incubator at 34C. All 12 combinations gave high survival (90 or 100%) for three days but only one method (wooden cage, with attendants, honey) gave 100% survival to day seven. Factors affecting queen survival were analysed. Across all combinations, attendant bees significantly increased survival (18% vs. 53%, p<0.001). In addition, there was an interaction between food type and cage type (p<0.001) with the honey and plastic cage combination giving reduced survival. An additional group of queens was reared and held for seven days using the best method, and then directly introduced using smoke into queenless nucleus colonies that had been dequeened five days previously. Acceptance was high (80%, 8/10) showing that this combination is also suitable for preparing queens for introduction into colonies. Having a simple method for keeping newly-emerged virgin queens alive in cages for one week and acceptable for introduction into queenless colonies will be useful in honey bee breeding. In particular, it facilitates the screening of many queens for genetic or phenotypic characteristics when only a small proportion meets the desired criteria. These can then be introduced into queenless hives for natural mating or insemination, both of which take place when queens are one week old.

## Introduction

Beekeepers and researchers often keep honey bee queens alive outside a colony for short periods of time. For example, queens are frequently sent through the mail from a queen breeder to another beekeeper. In this situation the queen is generally mated and spends a few days in the cage and is then introduced into a queenless hive. A recent paper from Gençer [1] highlighted the need for mated queens in times of the year when queen rearing is not possible, and devised a successful methodology to overwinter them in reservoir colonies in order to have queens available in early spring. Virgin queens may also be held outside a colony as part of the queen rearing process, allowing a greater flexibility in the schedule. Commercial queen rearing typically produces a sequence of mated queens from each mating nucleus hive and if the mating flights of the current batch of queens are delayed due to poor weather, the queen cells can be emerged in an incubator and the resulting virgin queens would be introduced into the mating hives instead of ripe queen cells. This methodology can provide beekeepers with additional time, up to approximately one week [2].

Another advantage of emerging and keeping virgin queens out of a colony is that it provides an opportunity for selection and testing. Selection can be very simple and quick, such as when a queen is visually inspected for wing deformities or appropriate body colour, or more technical, such as when wing morphometry or genetic tests are performed [2]. For example, when selecting for certain behavioural traits like hygienic behaviour, many virgin queens from a hygienic colony can be reared, then genotyped using a small piece of wing tissue [3] in order to identify queens that have the same father as the workers that are most hygienic [4]. With the progress being made in honey bee genetics, including genome sequencing [5] and the identification of genes [6] and quantitative trait loci [7]–[11] linked with behavioural or other phenotypical traits, it is likely that in the future molecular markers that denote desirable characteristics will be available as tools for marker-assisted selection.

One challenge in using intra-colony selection with molecular markers is that the majority of queens will often be discarded. If, for example, behavioural tests on the workers in a hygienic colony show that only one or two patrilines are hygienic [4], then only approximately 10% of any queens reared will belong to these patrilines given that honey bee queens mate with multiple males [12]–[14]. If every daughter queen has to be held in a colony while testing is being carried out, rather than held in a cage outside a colony, this will require much greater resources, effort and cost.

Therefore, there is an incentive to streamline the process to find the most economical method to keep a majority of queens alive for the greatest number of days.

Nelson and Roberts [15] report that of 12 virgin queens stored in bespoke wooden cages, one died after three days, one after five days and five after 17 days. The five surviving queens were artificially inseminated and introduced into colonies on day 17. The purpose of our study was to investigate the effects of three factors (cage type, food type, presence or absence of attendant workers) on the survival of newly-emerged virgin queens stored in commercially available mailing cages. We measured survival for the first week of life because this is a biologically relevant duration given that natural mating [16]–[19] occurs approximately one week into adult life. Additionally, one week is also the optimum time for instrumental insemination [20], [21]. Lastly, one week is sufficient time to carry out genetic tests, which may involve a few days delay to deliver samples to a testing lab. Our results show that the different combinations of factors gave very different survival rates, ranging from 0% to 100% after one week. Only one method (wooden cage, honey, attendants present) gave 100% survival at day seven.

## Methods

### 1. Rearing and preparation of virgin queens

Queen cells were reared using standard beekeeping methods in which one-day-old larvae from worker cells were transferred (“grafted”) into queen cups and reared in queenless colonies [19]. The hives used were in the apiary adjacent and belonging to the laboratory. The bees were of mixed European races, predominantly *Apis mellifera mellifera*. Ten or eleven days after grafting, sealed queen cells were placed individually into glass vials in an incubator (34C) and were kept there until emergence, after which they were placed in cages.

### 2. Conditions under which queens were held

Queens were held individually in cages in a temperature controlled room, 22C and given water *ad libitum* by placing droplets on the mesh covering each cage. Survival was determined at 0900 and 1800 each day. Three factors (cage type, food type, attendant workers) were tested in a complete three-way design, with 12 combinations and 10 queens per combination.

#### Cage type

**Figure 1:**
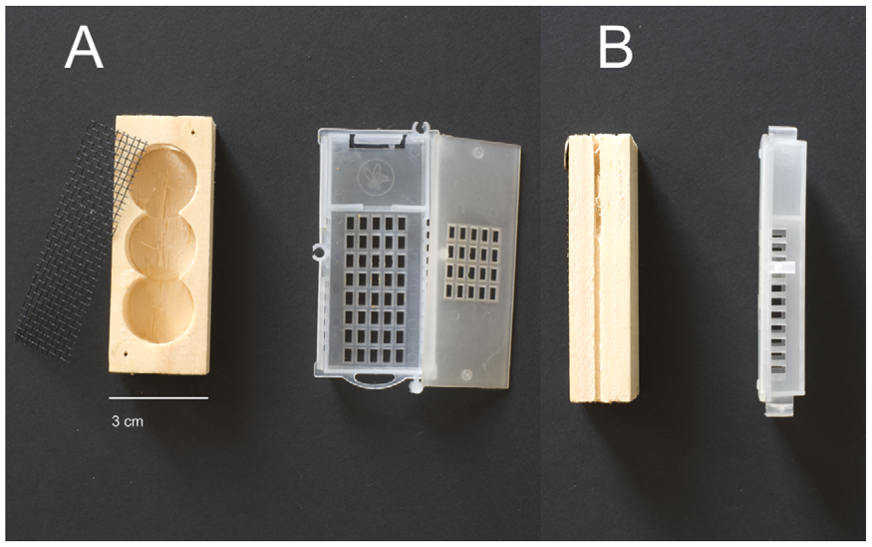
Cage types used in the experiment, top (A) and side (B) view. “Three-hole” wooden cage (left) and “Puzzle” plastic cage (right).

(2 treatments: wooden cage, plastic cage). We used two commercially-available and commonly-used queen mailing cages, one made of wood and one of plastic: “Three-hole” wooden cages with a metal mesh top (manufacturer W. T. Kelley, USA) and plastic “Puzzle” cages (manufacturer Swienty A/S, Denmark) (Fig 1).

#### Food

(3 treatments: candy, honey, candy+honey). Beekeepers have various preferences for feeding queens in cages. Queen cage candy is widely used, especially when sending queens by mail, because it is solid. Honey is natural and although it is not suitable for use when mailing queens, it is suitable for keeping queens alive if mailing is not needed. Candy was prepared by mixing together semi-crystallised honey and powdered sucrose in an approximate 1:4 weight ratio. Each cage was given 0.5–0.8 g honey in one of the end holes of the cage, or 8–10 g of candy by filling the food compartment in a plastic cage or one of the three circular cavities in a wooden cage, or both. Whenever honey was used, either alone or in combination with candy, more was given when daily inspections showed that existing supplies were low. The honey used was from our own apiary and so was known to be free of American foulbrood spores, as this disease is extremely rare in Britain and has never occurred in our apiary. We recommend that where AFB is common, honey should either not be used or, if practical, first be sterilized with γ-rays.

#### Attendant workers

(2 treatments: 5 or 0 workers) A frame containing sealed brood was placed in the incubator. Five newly-emerged workers were collected and introduced in each cage.

### 3. Survival of queens and introduction in nucleus hives

Following the experiment that measured cage survival, we reared ten additional queens and held them for one week using the method that gave the greatest survival (wooden cage, honey, five attendants) to test their acceptance into queenless nucleus hives. These were five-frame medium depth Langstroth with two to three frames of bees including one to two with brood, and were fed twice with 300 ml of 2M sucrose solution prior to and during queen introduction. Queens were introduced using the direct method with smoke [22] five days after queen removal, which maximises acceptance rate. We determined the acceptance of each introduced queen 24 h later by inspecting the hive. Any hive in which the queen was not seen was closed and checked again after one hour to be verify it was absent.

### 4. Statistical analysis

Data were analysed in R 2.10.1 [23] by fitting a linear generalised mixed-effects model using the LMER function of the LME4 package [24]. We included colonies from which the larvae used to rear queens were collected as a random effect to control for the non-independence of the data [25]. For model selection, we used the protocol proposed by Zuur et al. [25, chapter 5]. To explore the best random effects structure, we compared random intercept models with random intercept and slope models [25].

We tested the significance of fixed effects (food, cage, attendants) on queen survival at day seven by treating queen survival as a binomial response: 0 for queens that died and 1 for queens that survived. Additionally, queen weight at emergence was entered as a covariate. We used the Wald test to determine the significance of each fixed effect and the likelihood ratio test was used to test for significant interactions [25]. Since food treatments had three levels we performed pairwise comparisons using the multicomp package [26] and corrected significance levels with sequential Bonferroni [27]

## Results

### Queens obtained

In total 120 virgin queens were obtained from larvae that were transferred from six “mother colonies” (14 queens obtained from colony A, 17 from B, 53 from C, 6 from D, 19 from E and 11 from F). We randomly allocated 10 queens to each of the 12 combinations.

### Queen survival in cages

All 12 combinations initially performed well with 90% or 100% queen survival up to day three (Table 1). However, by day 7 survival varied from 0 to 100% with only one combination (wooden cage, honey, with attendants) achieving 100% survival (10/10 queens alive; Table 1).

**Table 1:** Survival of virgin queens during the first seven days of adult life under specified conditions.

	Conditions:		Number of virgin queens alive after experimental day:						
Attendants	Cage	Food	1	2	3	4	5	6	7
0	Plastic	Candy	10	10	10	10	5	1	1
5	Plastic	Honey	10	10	10	10	6	2	1
0	Plastic	Honey	10	9	9	4	1	1	0
0	Wood	Candy	10	10	9	9	7	6	4
5	Wood	Candy	10	10	10	10	9	9	4
0	Wood	Honey	10	9	9	9	8	7	3
0	Plastic	Honey+Candy	10	10	9	6	3	2	2
5	Plastic	Candy	10	10	10	10	9	8	7
5	Plastic	Honey+Candy	10	10	10	10	10	10	6
5	Wood	Honey+Candy	10	10	10	9	9	9	4
0	Wood	Honey+Candy	10	10	9	8	6	3	1
5	Wood	Honey	10	10	10	10	10	10	10

**Figure 2:**
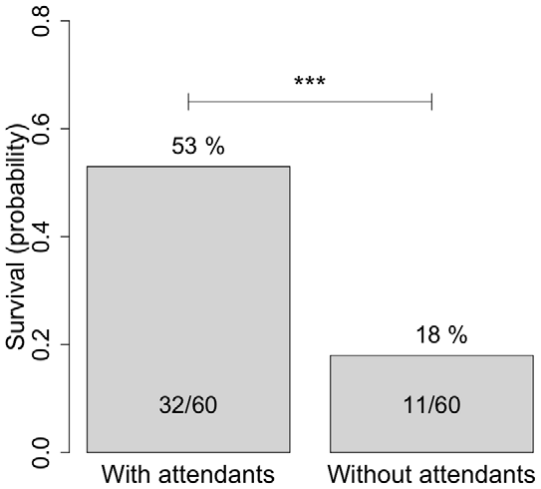
Probability of surviving until day 7 of virgin queens in cages with and without attendants, across the other treatments.

To analyse queen survival we started with a model containing all the effects and interactions and then removed the non-significant interactions [28]. The presence of attending bees was an important factor, increasing mean survival probability by 35% across all combinations (Figure 2; survival without attendants  = 18%, with attendants  = 53%, z = 3.743, *p*<0.001).

**Figure 3:**
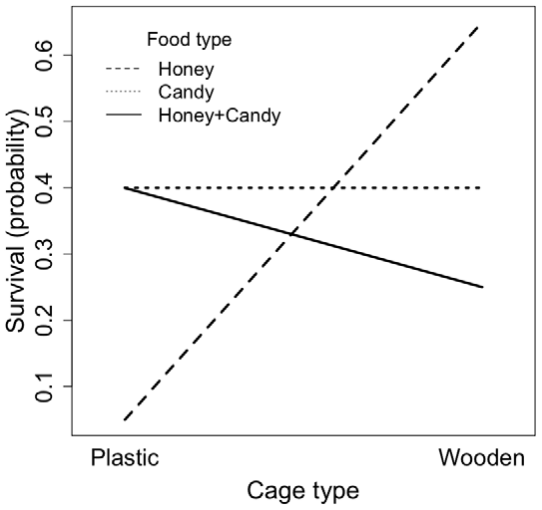
The interaction between cage and food type shows that honey alone negatively affects queens' survival in plastic cages.

We found a significant interaction between food type and cage type (Figure 3; LRT  = 25.352, *df* = 2, *p*<0.001). Because of this interaction, we analysed the effect of food separately in plastic and wooden cages. In plastic cages, honey alone had a negative impact on survival compared to candy alone, reducing survival by 35% (z =  −2.588, *p* = 0.0206). Conversely, using honey with candy improved the survival duration by 35% compared to honey alone (z = 2.704, *p* = 0.0206). The difference between candy alone or candy with honey was not significant (z = 0.215, *p* = 0.8314).

In wooden cages the differences in survival duration among food types, (proportion alive after 7 days: candy 40%, honey 65%, both 25%), were not significant when comparing honey and candy (z = 1.224, *p* = 0.4418) and honey and candy with candy alone (z = −1.208, *p* = 0.4418). However using honey with candy instead of honey alone showed a tendency to reduce survival (z =  −2.305, *p* = 0.0636).

There was no effect of queen weight on survival (z = −0.484, *p* = 0.6282).

### Acceptance of queens into queenless nucleus hives

Eight of ten (80%) queens were successfully accepted into the queenless nucleus hive. This proportion is not significantly different from the 100% acceptance rate previously reported by Pérez-Sato et al. [22], (*p* = 0.1082, chi-square test).

## Discussion

Our results show clearly that there is significant variation in the survival of virgin queens under different combinations of cage conditions during their first week of adult life. The best combination (wooden cage, honey, with attendant workers) gave 100% (10/10) survival in experiment 1 and also 100% in experiment 2 (10/10). The worst combination (plastic cage, honey, without attendant workers) gave 0% (0/10) survival.

We suggest the following reasons why certain combinations resulted in low survival. Plastic cages are designed to use candy not liquid honey. Even the small amount of honey we placed in the cage resulted in the queen becoming covered, which presumably hastened death. However, in the wooden cages the queens did not get covered presumably because the honey was partly absorbed into the wood. Attendant workers were the most important factor increasing queen survival by 35%. Therefore we highly recommend that they should be provided. Although an important goal of our project is to find a method that minimises the workload needed to maintain queens alive, the additional work needed to provide attendants is not great. It takes just a few minutes per cage, and workers are easily obtained either from a colony or by emerging brood in an incubator.

The only method to give 100% 7-day survival is simple to use and also gave high acceptance, 80%, of queens introduced into queenless colonies. This was not significantly lower than the 100% acceptance measured in a previous study using the same introduction method [22]. As a result, we are confident in recommending this method to beekeepers and scientists who are rearing and breeding queens as it provides similar survival rate to previous studies [1] but uses commercially available materials.

With the progress being made in honey bee genetics it is likely that in the future, there will be greater use of marker-assisted selection (MAS) on queens to decide which to retain in a breeding program or provide to beekeepers. Genetic tests can be made within a few days, and individual queens kept or discarded according to the results. A honey bee queen's one week pre-mating (or pre-insemination) period is an ideal time to perform tests as each queen can be kept in a cage, rather than in a colony, thereby saving time, effort and hive resources when compared to the alternatives, such as keeping queens in hives or in queen “banks”. Queen banking, in which caged queens are held in a populous and well-fed queenless colony, is often used by commercial queen rearers to keep mated queens alive for a few days or weeks prior to being sold. Virgin queens can also be kept alive in queen banks prior to insemination [21], [29]. However, based on our own experience (Ratnieks, personal observation), virgin queens have poor survival when banked compared to mated queens.

Queen rearing has been practised for over 100 years using the same basic method developed by Doolittle in the USA, involving the transfer one–day-old larvae. Queen breeding has benefited from the development of instrumental insemination, and will likely soon see benefits coming from the use of molecular markers (MAS) for desirable characteristics. But to use these methods effectively they will need to be combined with modifications of traditional procedures, such as keeping queens alive in cages.
